# Magnitude of Intention to Leave and Associated Factors among Health Workers Working at Primary Hospitals of North Gondar Zone, Northwest Ethiopia: Mixed Methods

**DOI:** 10.1155/2019/7092964

**Published:** 2019-07-16

**Authors:** Nigusu Worku, Amsalu Feleke, Ayal Debie, Adane Nigusie

**Affiliations:** ^1^Debretabor Health Sciences College, Debretabor, Ethiopia; ^2^Department of Health Systems and Policy, Institute of Public Health, College of Medicine and Health Sciences, University of Gondar, Gondar, Ethiopia; ^3^Department of Health Promotion and Behavioral Sciences, Institute of Public Health, College of Medicine and Health Sciences, University of Gondar, Gondar, Ethiopia

## Abstract

**Background:**

Human resource is the most crucial resources for the survival of an organization. Intention to leave is an employee's plan to leave their current job in the near future and is used as a proxy indicator for measuring turnover in cross-sectional surveys. In developing countries human resource shortages are not only due to production of health professionals but also because of employee turnover and instability at health facilities.

**Objective:**

This study aimed to assess the magnitude of intention to leave and associated factors among health workers working at primary hospitals of North Gondar Zone, Northwest Ethiopia.

**Methods:**

Institution based cross-sectional mixed methods' (both quantitative and qualitative) study design was conducted among health workers working at primary hospitals of North Gondar zone. Self-administered standardized structured questionnaires for quantitative and interview guide for qualitative were used for data collection. Variables having p-value less than 0.2 during bivariable analysis were entered into multivariable logistic regression model. Thematic analysis was done for qualitative data analysis.

**Results:**

A total of 382 health workers were participated in the study with a response rate of 93.6%. Overall, 67.8% of them were intended to leave their current organization. Age of participants, 20-29 years (AOR=3.96; 95%CI: 1.04, 15.07), living out of family (AOR= 1.73; 95% CI: 1.23, 3.02), opportunity of other job (AOR= 2.04; 95% CI: 1.21, 3.45), performance appraisal system (AOR= 2.97; 95%CI: 1.64, 5.36), and affective commitment (AOR= 3.12; 95% CI: 1.64, 5.92) were the factors affecting health workers intention to leave current organization.

**Conclusion:**

overall, magnitude of health workers intention to leave their current organization was high. Therefore, healthcare managers, supervisors, and policymakers need to develop and implement retention strategies that aim to improve the retaining of healthcare workers at their working organization such as unifying healthcare providers who are living separately with their families, use evidence-based performance evaluation mechanism, and make efforts to develop a sense of ownership in the health workers, which will reduce health professional's intention to leave their organization.

## 1. Background

Intention to leave is an employee's plan to leave their current job in the near future [1] and is used as a proxy indicator of turnover in cross-sectional surveys [2]. Out of fifty-seven countries experiencing critical shortage of human resource for health in the world, thirty-six are located in Africa; particularly it is the most severe in Sub-Saharan African countries [3]. Health workers turnover is an increasing problem that threatens the function of the health care sector worldwide, especially in developing countries [4]. In developing countries human resource shortages are not only due to production of health professionals, but also because of employee turnover and instability at health facilities [5]. The magnitude of intention to leave across African countries was from 18.8 to 41.4% [6] and in Ethiopia from 50 to 83.7 % [7–9].

The WHO recommendation of health workers to population ratio was a minimum of 2.5 health workers per 1000 population [4]. Large population has left with low health work force density of 0.84 per 1,000 people in Ethiopia even though it has the highest number of health workers in Sub-Saharan Africa [10]. The insufficient numbers, skill imbalances, mal distribution, low motivation, and poor performance of health workers compromise the delivery and expansion of priority health programs in low and middle-income countries [11, 12]. Studies in Taiwan and Ghana showed that job satisfaction, salary, and promotion were the factors affecting health workers intention to leave [13, 14]. Factors such as health care providers profession, salary, work experience, job satisfaction, organizational management, working environment, inadequate payment, and poor educational development were affecting health professionals' intention to leave their current working organization in Ethiopia [7, 8, 15].

Shortage of trained health workers, uneven distribution of health workers between urban and rural areas, underproduction of high and mid-levels of trained personnel, and low retention, including “brain drain” of health workers to more developed countries, are characteristics of human resources for health crisis in Ethiopia [16].

In 2015, the Health Sector Transformation Plan (HSTP) of Ethiopia showed that equity in healthcare provision and ensuring availability of best healthcare to all is the first among the four agendas [17]. However, this could be delivered when health care providers are committed, experienced, motivated, and stable in their work place. Therefore, this study aimed to assess the magnitude of intention to leave and associated factors among health workers working at primary hospitals of North Gondar zone, Northwest Ethiopia.

## 2. Methods and Materials

### 2.1. Study Design and Setting

**Figure 1:**
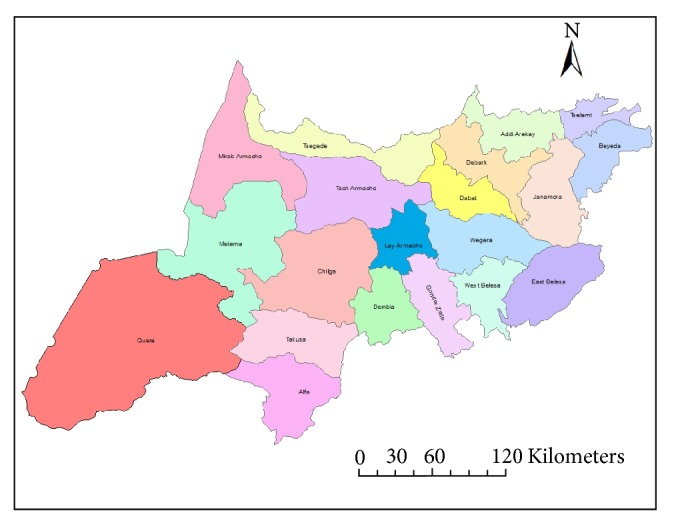
Map of Districts of North Gondar zone in Amhara National Regional State, Northwest Ethiopia, 2017.

Institution based cross-sectional mixed methods' (both quantitative and qualitative) design was conducted among health workers working in North Gondar zone primary hospitals from March 15 to April 30, 2017. The study was conducted in North Gondar Administrative zone which is one of the zones of Amhara national regional state, Northwest Ethiopia. According to 2007 Central Statics Agency (CSA) report the zone had a total population of 2,929,628 [18]. Accordingly, the Human Development Index (HDI) for Amhara National Regional State was 0.525[19]. There were nine woredas and one town administration in the zone. There were around 543 healthcare professionals working at nine public primary hospitals. Among these healthcare providers, 338 nurses, 75 pharmacists, 56 medical laboratories, 34 medical doctors, and 40 other health professionals were working in the nine hospitals. Additionally, the zone has a total of 24 districts, 126 health centers, and 573 health posts (Figure 1).

### 2.2. Population and Sampling Procedure

The source and study populations were all health workers who were working in primary hospitals of North Gondar zone, Northwest, Ethiopia. The sample size was determined by using single population proportion formula considering an assumption of magnitude of intention to leave their current organization (59.4%) [7], 5% margin of error, 95% confidence level, and 10% nonresponse rate. As a result, the final sample size was 408. Then, the sample was proportionally allocated to each primary hospital based on the number of health workers who are working in these hospitals. Finally, simple random sampling technique was used to select participants by using their payroll as sampling frame.

Qualitative data were collected through key informant interview. Four of nine primary hospitals in North Gondar zone were selected for qualitative data collection sites because they are located in different geographical locations which are Debark from high land* (Dega), *Guhalla and Metema from low land* (kola), *and Ayikel from* (Woina Dega)*. Purposeful sampling was used to select the sites and recruit key informant interview participants in the selected hospitals. Nine key informants were selected purposefully considering the key informant's knowledge of the working environment, interaction with the staffs and the communities, and working experiences on their current organization. As a result, among these nine key informants 2 chief executive officers, 2 medical directors, 2 head nurses, 1 head of medical laboratory, 1 head of pharmacy unit, and 1 more experienced nurse were selected purposely. The distribution of key informants among hospitals was Debark (3), Metema (2), Guhalla (2), and Ayikel (2).

### 2.3. Measurements

Intention to leave is the intention of an employee to leave their current working organization in the near future and measured by using three item questions with a 5 point Likert scale and participants scored more than 60% of the total score of intention to leave measuring score was considered as having intention to leave their current working organization [20]. Accordingly, affective, continuance, and normative commitments of health professionals towards their organization were measured by using three item questions with 5-point Likert scale for each commitment domains. As a result, participants who scored more than 60% of the total score of each commitment measurement score were considered as having high affective, continuance, and normative commitments, respectively.

### 2.4. Data Collection Tools and Procedure

Quantitative data were collected using standardized self-administered structured questionnaire, adopted from different literatures [8, 21]. However, semistructured interview guide was used to collect qualitative data. Socioeconomic characteristics, job satisfaction, organizational commitment, and health workers intention to leave their current working organization were measured in this study.

Interview guide was used for qualitative data collection through probing of the key informants following the information they had been provided. The average time taken for conducting each interview was 30 minutes.

### 2.5. Data Quality Control

Three diploma clinical nurse data collectors and two BSc nurse supervisors were recruited and one day training was given for data collectors and supervisors. The questionnaire was first prepared in English and translated to Amharic then back to English in order to ensure its consistency. Pretest was conducted among 41 health workers at Addis Zemen primary hospital and some basic modification has been done based on pretest findings. The internal consistency for each dimension of the questionnaire was checked through calculating Cronbach's alpha. The Cronbach's alpha for each dimension was greater than 0.7, particularly Cronbach's alpha value for the outcome variable was (*α*= 0.82). The qualitative data was collected by investigators after debriefing key informants, arranging favorable time, and place for interviewee. One note taker and tape recorder, as well as one interviewer, were involved in the interviewing process. Consequently, the audio recording was transcribed in Amharic and then translated to English and finally back to Amharic to ensure the accuracy and consistency of the collected information.

### 2.6. Data Management and Analysis

The data were checked for completeness then, entered into Epi-info version 7.2, and exported to SPSS version 20 software for quantitative data analysis. Descriptive statistics such as frequencies and percentage has been presented using graphs and tables. Binary logistic regression model was used to identify the potential predictor variables for health workers intention to leave their current organization. Those independent variables which had p-value of less than 0.2 during binary logistic regression analysis were entered during multivariable logistic regression analysis. Then, Adjusted Odds Ratio (AOR) with 95% CI and P-value <0.05 were used to identify factors significantly associated with intention to leave the organization. Hosmer and Lemshow goodness of fit test was used to check the model fitness. The qualitative data were transcribed from audio recordings in Amharic, translated into English, and backtranslated into Amharic to ensure accuracy of the translation. The data were thematized by participant's type of professions and analyzed each hospital's responses in a framework. The investigators refined the themes, found commonalities, and wrote up the findings supplementing with the quantitative findings.

### 2.7. Ethics Approval Statement

Ethical clearance was obtained from the Ethical Review Committee of Institute of Public Health, College of Medicine and Health Sciences, University of Gondar. Permission letter also was obtained from Amhara National Regional Health Bureau and the respective hospitals. Written informed consent was taken from each participant. Each eligible study participant was informed about the purpose and importance of the study. Participants had been gotten the assurance that their name was not written on the questionnaire and confidentiality of the data kept at all levels.

## 3. Results

### 3.1. Sociodemographic Characteristics

A total of 382 health workers were participated in the study with a response rate of 93.6%. The age of participants was from 20-55 years with median age of 26 with interquartile range of 4.8 years. Almost half of the participants were unmarried and more than fifty percent (57.3%) were males. Nearly half of the participants were Bachelor of Science (BSc) health science graduate professionals. More than two-thirds (69.3%) of the participants had less than five years' experience and their median monthly salary was USD 159 (ETB 4,446.00). Additionally, more than sixty (62%) of participants who were working in the study area were nurse professionals (Table 1).

### 3.2. Organizational Commitment Related Factors

**Figure 2:**
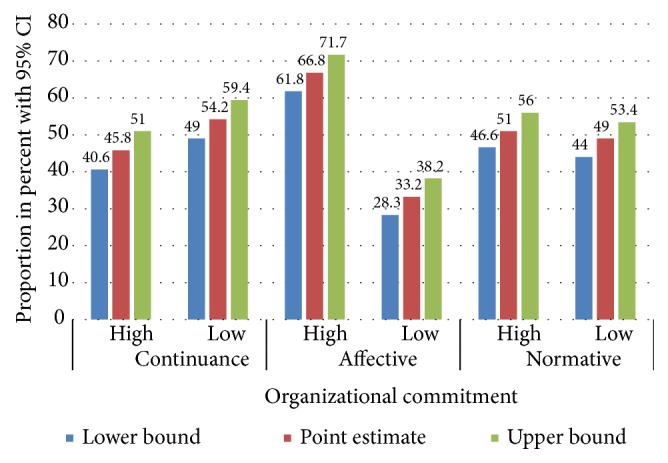
Organizational commitment among health workers working in North Gondar zone primary hospitals, 2017.

Health workers who were working in the study area had relatively high normative (51%) and affective commitments (66.8%) towards their working organization. However, the continuance commitment of participants towards their organization was low (45.8%) (Figure 2).

### 3.3. Magnitude of Intention to Leave and Job Satisfaction

Magnitude of intention to leave among health workers working at primary hospitals of North Gondar zone were 67.8%. About two-third (67.5%) of participants were satisfied with their coworker relationships. Furthermore, 63.9, 77.2, and 70.4% of respondents were unsatisfied regarding their performance appraisal, educational development, recognition, and rewarding in their current working organization, respectively (Table 2).

### 3.4. Factors Associated with Intention to Leave

Binary logistic regression model was applied to identify the potential predictor variables that affect health workers intention to leave their current organization. As a result, variables having p-value less than 0.2 during binary logistic regression analysis were entered in the multivariable logistic regression analysis model. Health workers whose age from 20-29 years were 3.96 times (AOR: 3.96; 95% CI: 1.04, 15.07) more likely intended to leave their current working organization than those health workers whose age greater than 40 years. Those study participants who lived separately from their family were 1.73 times (AOR: 1.73; 95% CI: 1.23, 3.02) more likely intended to leave their current organization compared to those who are living with their families. A 32-year-old pharmacy technologist said “I didn't want to leave this hospital even if I am not satisfied with the overall working system of the hospital. Because I am living together with my wife and children. If I want to leave this hospital, I will expense extra costs.”

Health workers who have an opportunity of getting other works were 2.04 times (AOR: 2.04; 95% CI: 1.21, 3.45) more likely intended to leave their current organization as compared to those who did not get other works. The in-depth interview finding showed that key informants who are laboratory and pharmacy professionals reported that “currently professionals in our departments are not easily available in the market. This results high market demand and gives a good chance to leave our current job and getting other jobs easily.”

Health workers who were unsatisfied with their performance appraisal results were 2.97 times (AOR 2.97; 95% 1.64, 5.36) more likely intended to leave their current organization as compared to those participants who are satisfied with their performance appraisal. A 27-year laboratory technologist said “What I have observed from all my seven years work experience was that there is lack of fair performance evaluation system. It is a very poor system and the criterion has nothing to do with meritocracy. This makes me disappointed and dissatisfied. How much should I bear the burden and I decided to leave my current job as soon as possible.”

Health workers who have low affective commitment were 3.12 times (AOR 3.12; 95% CI: 1.64, 5.92) more likely intended to leave their current organization as compared to those who have high affective commitment. A 36-year-nurse said “I am not satisfied with my current working organization because there was no professional development, recognition, incentives/ rewards, adequate working materials, good working environment and vibrant leaders. This makes me to dissatisfy and intended to leave my current working organization” (Table 3).

## 4. Discussion

This study revealed that the overall magnitude of intention to leave among health workers working at primary hospitals of North Gondar zone was 67.8% (95% CI: 63.4, 72.3). This finding was in line with studies done in Ghana (69%) [14] and Jimma zone, Ethiopia (63.3%)[15]. However, it is higher than the studies conducted in University of Gondar referral hospital (52.5%) [22], North Shoa zone (61.3%)[23], East Gojjam zone (59.4%)[7], Sidama zone (50%)[8], Tanzania (18.8 %), Malawi (26.5%), South Africa (41.4%) [6], Iraq (55.2%) [24], Switzerland (16.7%)[25], Brazil (22.1%)[26], and Japan 57.8% [27]. The possible explanation might be the difference in the study area, period and design. This might be due to the study includes only health professionals who are working at primary hospitals and the study area might have poor infrastructures and enforces health professionals to leave their current organization. On the other hand, this finding is lower than a study conducted in Hawassa referral hospital (83.7%) [9]. This variation might be due to differences in study population. In the previous studies the only study participants were nurses and nurses might have high work load and this enforces them to be more intended to leave their working organization as compared with other health workers.

Health workers aged 20-29 years were 3.96 times more likely intended to leave their current working organization as compared to health workers aged greater than 40 years. This finding is consistent with studies done in Sidama, Ethiopia, and Iraq [8, 24], 12 countries of Europe [28], retention priorities of intergenerational nurse work force forum report [29], and causal model of turnover [1]. This might be due to young health workers might be unmarried that makes them to move anywhere in anticipation of getting better benefits. Additionally, young health professionals are more exposed to repetitive tasks, participating less in decision making, lacking knowledge of their work, being paid less, and having fewer close friends in the workplace. These factors, they argued, could contribute to greater dissatisfaction with the organization among the youngest age groups. Our finding contradicts with the finding from Brazil [26]. This variation might be due to the study in our context was focused on intention to leave all health professionals working organization, but in Brazil the study emphasized on intention to leave the profession specifically for nurses. Since the nature of nurse profession had high workload, they might change their profession.

Health workers who lived out of their families were 1.73 times more likely intended to leave their current organization as compared to those who are living with their families. This finding is in line with a study conducted in East Gojjam zone health institutions [7]. The possible explanation might be health workers who lived separately from their families might be suffered by living cost and lacks stability. This might make them to prefer living with their families for maintaining their stability and for reducing their living cost.

Health professionals who have chance of getting other job opportunity were 2.04 times more likely intended to leave their current organization as compared to those who did not get other job opportunity. This result is in line with studies conducted in Iraqi [24]. This might be due to some health professionals having high market demand and probably they thought that they may get better work setting.

Similarly, health workers who have been unsatisfied with the organization performance appraisal system were 2.97 times more likely intended to leave their current organization than who have satisfied by performance appraisal system. This result is in line with a study done in Oromia region and public hospitals of West Shoa zone [30, 31]. The reason may be due to unfair performance evaluation system is currently used as one of the criteria for getting professional development or graduate training. Participants who have low organizational affective commitment were 3.12 times more likely intended to leave their current working organization as compared to their counterpart. This finding is supported by a meta-analysis report on intention to leave [21] and an empirical study in Istanbul, Turkey [32]. This might be due to employees who feel sense of belongingness might involve and linked emotionally with the organization goals and strategies and might results in intended to stay their current organization. According to the literature, the most popular and thoroughly multidimensional model of organization commitment [33] includes affective, normative, and continuance components, all of which are thought to contribute to employee retention.

Healthcare providers whose household monthly income from USD 113-146 were less likely intended to leave their current working organization as compared with health workers whose household monthly income USD ≥ 190 by 57%. The purchasing parity index for Ethiopia according to 2018 report ranged 1.5-2.7 [34]. This Big Mac Index indicated that the Dollar was overvalued which means the purchasing power parity of Dollar in Ethiopia is higher than in United States. As a result, those participants who have high monthly household income might have higher intention to leave because of the increasing their need to live in urban areas for improving their living conditions.

### 4.1. Strengths and Limitations of the Study

This study assessed health workers intention to leave their current organization among all health workers working at primary hospitals. Furthermore, the find was supplemented with qualitative to support the quantitative findings. However, the study might be prone to response bias as a result of using self-administered questionnaire. It did not also show the cause-effect relationship because of its cross-sectional nature and it does not measure directly the actual health workers turnover. Additionally, the study focuses on intention to leave the organization, but not measure other aspects of intention to leave, such as intention to leave professional careers, public organizations, and rural areas.

## 5. Conclusion

Overall, the magnitude of intention to leave among health workers working in North Gondar zone primary hospitals was high. Most of the health workers were satisfied with coworker relationship within their organization, but organizational policy, performance appraisal, educational opportunity, supervision, payment, and benefit mechanism were reported dissatisfying by most of health workers. Age of health workers 20-29 years, living out of family, job opportunity, poor performance appraisal, and low affective commitment were the factors affecting intention to leave. Therefore, health care managers, supervisors, and Health care policymakers need to develop and implement retention strategies that aim to improve the retention of health care workers at their working organization such as unifying healthcare providers who are living separately with their partner (families), use evidence-based performance evaluation mechanism, and make efforts to develop a sense of ownership in the health workers. Researchers shall conduct a follow up study in order to measure the magnitude of the actual turnover among health workers. It is also better to assess health workers intention to leave professional careers, public organizations, and rural areas.

## Figures and Tables

**Table 1:** Sociodemographic and economic characteristics of health workers, Northwest Ethiopia, 2017 (n=382).

Variables	Category	Frequency	Percent (95%CI)
Sex	Male	219	57.3(51.6-62.4)
	Female	163	42.7(37.6-48.4)
Age in years	20-29	298	78.0(73.6-82.2)
	30-39	67	17.5(13.9-21.7)
	≥40	17	4.5(2.5-6.5)
Educational status	Diploma	181	47.4(42.7-53.0)
	Degree and above	201	52.6(47.0-57.3)
Marital status	Single	190	49.7(44.4-54.8)
	Married	192	50.3(45.2-55.6)
Family arrangement	Living with family	124	32.5(28.1-37.2)
	Living out of family	258	67.5(62.8-71.9)
Family dependency	Yes	118	30.9(26.4-36.1)
	No	264	69.1(63.9-73.6)
Position	Yes	56	14.7(11.1-18.1)
	No	326	85.3(81.9-88.9)
Experience in years	≤2	105	27.5(23.5-31.9)
	3 to5	160	41.9(36.9-46.3)
	≥6	117	30.6(26.4-35.1)
Monthly salary (USD)	≤ 112	119	31.2(26.3-35.6)
	113-146	68	17.8(14.1-22.1)
	147-189	88	23.0(19.1-27.5)
	≥190	107	28.0(23.6-31.9)
Profession	Nurse	238	62.3(57.3-67.0)
	Pharmacy	53	13.9(10.5-17.5)
	Medical Laboratory	39	10.2(7.2-13.6)
	Medical Doctors	24	6.3(4.2-8.9)
	Others	28	7.3(4.7-10.1)

USD: United States Dollar and CI: confidence interval.

**Table 2:** Job satisfaction by different dimensions among health workers Northwest, Ethiopia, 2017 (n=382).

Variables	Category	Frequency	Percent (95%CI)
Performance appraisal			
	Satisfied	138	36.1(30.9-41.4)
	Unsatisfied	244	63.9(58.6-69.1)
Educational development			
	Satisfied	87	22.8(18.8-26.5)
	Unsatisfied	295	77.2(73.5-81.2)
Recognition and reward			
	Satisfied	113	29.6(25.1-34.1)
	Unsatisfied	269	70.4(65.9-74.9)
Autonomy			
	Satisfied	180	47.1(41.9-52.6)
	Unsatisfied	202	52.9(47.4-58.1)
Payment and benefit			
	Satisfied	88	23.0(19.1-27.3)
	Unsatisfied	294	77.0(72.7-80.9)
Supportive supervision			
	Satisfied	150	39.3(34.2-44.2)
	Unsatisfied	232	60.7(55.8-65.8)
Workload			
	High	177	46.3(40.5-51.6)
	Low	205	53.7(48.4-59.5)
Coworker relationship			
	Good	258	67.5(63.1-72.3)
	Poor	124	32.5(27.7-36.9)
Organizational policy and Strategy			
	Satisfied	102	26.7(22.8-31.2)
	Unsatisfied	280	73.3(68.8-77.2)
Opportunity of other job			
	Yes	187	49.0(44.1-53.9)
	No	195	51.0(46.1-55.9)

CI: confidence interval.

**Table 3:** Factors associated with intention to leave among health workers in North Gondar zone primary hospitals, 2017(n=382).

Variables	Category	Intention to leave		COR (95%CI)	AOR (95%CI)
		Yes	No		
Age in years					
	20-29	201	77	3.07(1.28, 9.40)	3.96(1.04,15.07) *∗*
	30-39	41	26	1.86(0.76, 6.66)	2.73(0.78, 9.64)
	≥40	17	20	1	1
Performance appraisal					
	Satisfied	73	65	1	1
	Unsatisfied	186	58	2.86(1.83, 4.46)	2.97(1.64,5.36) *∗*
Educational opportunity					
	Yes	53	34	1	1
	No	206	89	1.49(0.90, 2.44)	1.38(0.71, 2.68)
Recognition & reward					
	Satisfied	69	44	1	1
	Unsatisfied	190	79	1.53(0.97, 2.43)	0.89(0.49, 1.61)
Family arrangement					
	Living with family	69	55	1	1
	Living out of family	190	68	2.23(1.42, 3.49)	1.73(1.23, 3.02) *∗*
Salary (USD)					
	≤ 112	82	37	0.79(0.44, 1.40)	0.99(0.40, 2.44)
	113-146	35	33	0.38(0.20, 0.71)	0.43(0.19, 0.95) *∗*
	147-189	63	25	0.89(0.47, 1.68)	1.13(0.43, 2.96)
	≥190	79	28	1	1
Supportive supervision					
	Satisfied	93	57	1	1
	Unsatisfied	166	66	1.54(0.99, 2.38)	1.11(0.61, 1.98)
Continuous commitment					
	High	110	65	1	1
	Low	149	58	1.52(0.99, 2.34)	1.45(0.85, 2.46)
Normative commitment					
	High	141	54	1	1
	Low	118	69	0.66(0.43, 1.01)	0.96(0.55, 1.65)
Affective commitment					
	High	155	100	1	1
	Low	104	23	2.92(1.74, 4.89)	3.12(1.64,5.92) *∗*
Opportunity of other job					
	Yes	146	41	2.58(1.65, 4.04)	2.04(1.21,3.45) *∗*
	No	113	82	1	1
Experiences in years					
	≤2	81	24	1	1
	3-5	104	56	0.55(0.31, 0.96)	0.90(0.45, 1.79)
	≥6	74	43	0.51(0.28, 0.92)	1.41(0.45, 4.43)

**∗**Significant at P-value <0.05, USD: United States Dollar, CI: confidence interval, COR: Crude Odds Ratio, and AOR: Adjusted Odds Ratio.

## Data Availability

All the data supporting the findings are within the manuscript. Additional detailed information and raw data are available from the corresponding author on reasonable request.

## Abbreviations

AC: Affective commitment
AOR: Adjusted Odds Ratio
ANRHB: Amhara National Regional Health Bureau
CC: Continuance commitment
CI: Confidence interval
COR: Crude Odds Ratio
CSA: Central Statics Agency
HDI: Human Development Index
HSTP: Health Sector Transformation Plan
OR: Organizational commitment
NC: Normative commitment.
